# Microfluidic-Based Oxygen (O_2_) Sensors for On-Chip Monitoring of Cell, Tissue and Organ Metabolism

**DOI:** 10.3390/bios12010006

**Published:** 2021-12-22

**Authors:** Mostafa Azimzadeh, Patricia Khashayar, Meitham Amereh, Nishat Tasnim, Mina Hoorfar, Mohsen Akbari

**Affiliations:** 1 Medical Nanotechnology & Tissue Engineering Research Center, Yazd Reproductive Sciences Institute, Shahid Sadoughi University of Medical Sciences, Yazd 89195-999, Iran; m.azimzadeh@ssu.ac.ir; 2 Stem Cell Biology Research Center, Yazd Reproductive Sciences Institute, Shahid Sadoughi University of Medical Sciences, Yazd 89195-999, Iran; 3 Department of Medical Biotechnology, School of Medicine, Shahid Sadoughi University of Medical Sciences, Yazd 89165-887, Iran; 4 Center for Microsystems Technology, Imec and Ghent University, 9050 Ghent, Belgium; patricia.khashayar@ugent.be; 5 Laboratory for Innovations in Micro Engineering (LiME), Department of Mechanical Engineering, University of Victoria, Victoria, BC V8P 5C2, Canada; mamereh@uvic.ca; 6 Center for Advanced Materials and Related Technologies, University of Victoria, Victoria, BC V8P 5C2, Canada; 7 Department of Mechanical Engineering, University of Victoria, Victoria, BC V8P 5C2, Canada; nishattasnim@uvic.ca; 8 Biotechnology Center, Silesian University of Technology, Akademicka 2A, 44-100 Gliwice, Poland

**Keywords:** oxygen sensors, microfluidics, organ-on-chips (OOCs), on-chip monitoring

## Abstract

Oxygen (O_2_) quantification is essential for assessing cell metabolism, and its consumption in cell culture is an important indicator of cell viability. Recent advances in microfluidics have made O_2_ sensing a crucial feature for organ-on-chip (OOC) devices for various biomedical applications. OOC O_2_ sensors can be categorized, based on their transducer type, into two main groups, optical and electrochemical. In this review, we provide an overview of on-chip O_2_ sensors integrated with the OOC devices and evaluate their advantages and disadvantages. Recent innovations in optical O_2_ sensors integrated with OOCs are discussed in four main categories: (i) basic luminescence-based sensors; (ii) microparticle-based sensors; (iii) nano-enabled sensors; and (iv) commercial probes and portable devices. Furthermore, we discuss recent advancements in electrochemical sensors in five main categories: (i) novel configurations in Clark-type sensors; (ii) novel materials (e.g., polymers, O_2_ scavenging and passivation materials); (iii) nano-enabled electrochemical sensors; (iv) novel designs and fabrication techniques; and (v) commercial and portable electrochemical readouts. Together, this review provides a comprehensive overview of the current advances in the design, fabrication and application of optical and electrochemical O_2_ sensors.

## 1. Introduction

Oxygen (O_2_) is one of the main components of cellular respiration and energy production [1]. The availability of O_2_ is a key metric that defines the pathway of adenosine triphosphate (ATP) generation and its resultant metabolites that serve as the living cell’s energy source [2,3]. In a high O_2_ environment, ATP is synthesized by the phosphorylation of the precursor molecule adenosine diphosphate (ADP). This process, thus called aerobic respiration, requires an adequate level of O_2_. It consists of the coupling of electron transport and oxidative phosphorylation, where O_2_ acts as the final electron acceptor from the oxidation of glucose and/or glycogen [4]. In low O_2_ environments, conversely, ATP is generated at an inefficient but rapid rate via a process called anaerobic glycolysis, where glucose and glycogen are metabolized to pyruvate and lactate in the absence of O_2_. This pathway is important in the functions of vital organs such as the kidney and retina as well as in tumor formation [5]. O_2_ availability determines the metabolic pathway that generates energy for cell function and survival and therefore is significantly important to measure for bioassays, cell culture and diagnostic applications.

Precise control of a small amount of fluid is possible inside microfabricated channels of microfluidic technology [6]. Integrated microfluidic chips are capable of performing highly sensitive and low-cost analyses. These platforms can be integrated with new technologies with cell culture/organoid studies at high temporal and spatial resolution. For instance, microfluidic platforms can quantitatively monitor cellular signals and cell secretions using well-developed cell-culture methods on microchips [7]. O_2_ measurement can be performed using sensors integrated into microfluidic chips for monitoring the metabolism and viability of cell, tissue, and organ [8,9]. Moreover, novel sensors embedded inside implantable microchips have recently been used for real-time in vivo oximetry in human or animal bodies [10]. Sensors can also be used to detect and quantify various analytes in a complex biological environment [11,12,13,14,15,16]. Therefore, a combination of integrated sensors is required for online and non-invasive monitoring of the cell intake, secreted metabolites, and microenvironment status for on-chip microfluidic studies such as OOC and bioreactors [12,13].

On-chip monitoring of O_2_ is pivotal in OOCs. The low concentration of O_2_ inside a small chip and its crucial biological role in cell metabolism and function would emphasize the need for its precise and selective quantification in the confined environment of a microfluidic channel or chamber [9,17]. With recent advances in microfluidics-based cell and tissue studies, such as OOC technologies, various sensors have been integrated into chips to monitor the microphysiological parameters of cells [12,18,19,20,21,22,23]. OOCs have the potential to better mimic human organs compared to the traditional in vitro models, and thus they can reduce the need for animal models in interventions such as the studies on drug efficacy and toxicity [24,25,26]. To model the function of many organs, such as the pancreas [27], brain [28,29,30], liver [31], vascular system [32], Gut [33], multiorgan approaches [34] and body-on-the-chip, OOC-integrated O_2_ sensing techniques have been used to culture cell monolayers, three dimensional (3D) cultures, spheroids, organoids and stem cells. They have also been used to model tumor microenvironment by mimicking the extracellular matrix (ECM) [35] and 3D culture of cancer cells [36,37].

Electrochemical and optical sensors are the main transducers for on-chip O_2_ monitoring [38], as they are precise, selective, and easy to miniaturize and implement inside chips [18,39]. In addition, micro and nanomaterials along with the innovative designs and polymers, and commercial readout devices are used for signal amplification to overcome the limitation related to the measurement of low O_2_ levels with electrochemical and optical sensors [13,40,41]. Here, we review the recent innovations in O_2_ sensors integrated into microfluidic chips, including OOC devices. We have categorized them based on their transducer type into two main sections, namely optical and electrochemical. We also discuss recent innovations and their advantages and disadvantages. Finally, we provide a comprehensive discussion of the current advances in the design, fabrication and application of optical and electrochemical O_2_ sensors.

Previous reviews have covered related topics such as oxygen control (2016) [9], general optical imaging and sensing (2014) [39], microfluidic OOC sensors (2010) [40], and other microphysiological sensors of OOCs [12,13,18,38]; however, there are no recent publications critically discussing the current advancements in OOC O_2_ sensing. The present review delves into a critical examination of recent developments in O_2_ sensors integrated into OOCs devices and provides a comprehensive comparison of their advantages, limitations and required future improvements.

## 2. Oxygen Sensors in On-Chip Systems

Methods of on-chip O_2_ measurement can be categorized into two groups of sensors: (i) optical and (ii) electrochemical. Here, we highlight the application of these two types of sensors in on-chip studies, explain their mechanism of action, and discuss their advantages and disadvantages. Table 1 represents the summary of advantages and disadvantages of optical and electrochemical methods for on-chip O_2_ measurement.

### 2.1. Optical Methods

Optical strategies for O_2_ sensing have been widely used in on-chip systems. Luminescent sensors are the most common type of these sensors that utilize either fluorescence or phosphorescence dyes that respond to O_2_ molecules as a quencher available inside the chip. Optical methods have been used more than electrochemical methods for on-chip O_2_ sensing due to their lower limit of detection ranges, lower cost and ease of manufacturing. They have great potential to be used as a portable point-of-need detection and connecting to commercial optical measurement (readout) devices. The advantage of the optical sensing technique is that O_2_ is not consumed during the optical measurement, unlike other methods. Additionally, optical fibers can measure O_2_ levels easier without the need of direct contact of the culture media by adding a protecting layer of oxygen diffusing materials, such as PDMS, on the optical fiber. This feature is beneficial for keeping the cell microenvironment intact or when O_2_ content or resource is limited. Optical O_2_ measurements can also be simultaneously performed with the measurement of other important parameters, such as pH and cell metabolites (such as lactate, glucose, and amino acids) [9,12,38,39].

In the following sections, we summarize the advancements in optical O_2_ sensors four main categories based on their type of innovations. Furthermore, to make the comparison easier, Table 2 represents the summary of most recent on-chip optical O_2_ sensors and their features, applied dyes, and their advantages.

#### 2.1.1. Basic Luminescence-Based Sensors

The luminescence-based sensing method relies on quenching of luminescence (fluorescence or phosphorescence) dyes by O_2_ molecules based on the Stern-Volmer equation [39], which reflects the relationship between the concentration of O_2_ and fluorescent intensity [52,53]. Dyes are mixed with polymers containing O_2_ permeable and soluble features to make a sensing layer on chip where the emission readout will be measured following an excitement in specific spectra. The main advantages of this strategy are its high sensitivity and selectivity toward O_2_, ease of fabrication, high photo-stability (suitable for long-term and continuous O_2_ monitoring), and short response time (typically less than a few seconds) [52,53,54].

The most popular dyes used for O_2_ sensing include metal–ligand complexes, particularly metalloporphyrins [39]. These dyes are photostable with sharp, strong luminescent signals that can be excited in the visible spectrum. Moreover, small changes in their structure (peripheral substitution) can alter their chemical and spectroscopic properties, suggesting that they are tunable. Due to the longer lifetime of their excitement, their quenching efficacy is high. Therefore, they have high O_2_ sensing sensitivity [39,44,55,56]. Few examples of the most used dyes are Platinum(II) tetrakis(pentafluorophenyl)porphyrin (PtTFPP) [57], Platinium (II) octaethylporphyrin (PtOEP) [42], Platinum (II)-meso-tetra(4-fluorophenyl)tetrabenzoporphyrin (PtTPTBPF) [43], Ruthenium-tris(4,7-diphenyl-1,10-phenanthroline) dichloride (Ru(dpp)) [55], and Singlet O_2_ sensor green (SOSG) [56]. In a few studies, other materials such as polymerized 2-hydroxyethyl methacrylate (HEMA) have been used as a dye [58].

**Figure 1:**
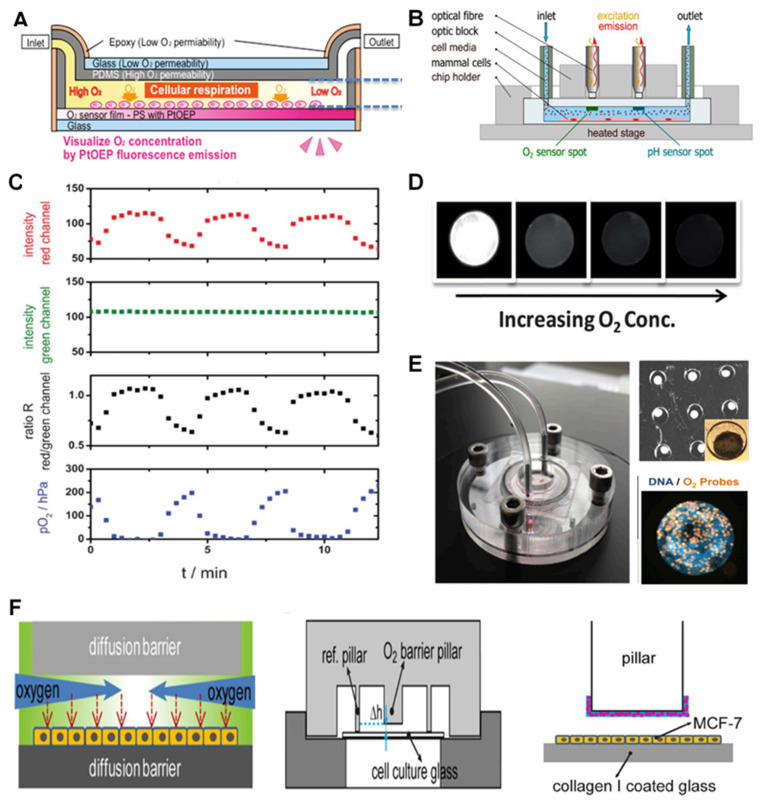
Basic luminescence-based sensors and microtechnology-based luminescent sensors for O_2_ monitoring in on-chip studies. (**A**) The concept of an integrated layer of O_2_-sensitive dye in an OOC device for liver studies. Reproduced with permission [42], copyright 2019, John Wiley & Sons. (**B**) Optical fiber-based detection of O_2_ and pH. Reproduced with permission [43], copyright 2021, Elsevier. (**C**) The ratiometric concept for optical O_2_ sensing through comparing the signals from O_2_-sensitive dyes with that of non-O_2_ sensitive ones as reference. Reproduced with permission [61], copyright 2013, The Royal Society of Chemistry. (**D**) PDMS Microbeads containing luminescent dyes were synthesized using microfluidics for O_2_ sensing. Their performance in an O_2_ gradient (different concentrations of O_2_) is represented. Reproduced with permission [68], copyright 2012, The Royal Society of Chemistry. (**E**) A bioreactor (liver-on-chip) device (left) for the analysis of drug effect on mitochondrial activity of the HepG2/C3A organoids. Each microwell contains an organoid (top right) with integrated microparticles with luminophores for imaging-based O_2_ sensing (right). Reproduced with permission [69], copyright 2016, National Academy of Sciences. (**F**) O_2_ gradient (left) inside the microbioreactor (down right) structure and the O_2_ barrier and sensing pillars with O_2_-sensing microbeads (pink circles) (right). Reproduced with permission [45], copyright 2017, Springer Nature.

The dyes are mostly embedded into dye-impregnated polymeric layers at the vicinity of the cell chambers during the fabrication of chips. The layers are developed by directly mixing or dissolving the dyes into the solvent or polymer and then forming a film layer inside or at the top of the microfluidic channels and chambers [59]. Impregnated polymers are then patterned into the cell chambers using spin coating [44], knife coating [60], and preheated coating dye solution [41]. Dyes are excited by either light-emitting diode (LEDs) or optical fibers. The signal readout is achieved by using Charge-Coupled Devices (CCD) camera [61], fluorescent microscope imaging [42] (Figure 1A) or via optical fibers [43] (Figure 1B). To quantify the signal in real-time, the collected optical signal is converted into an electrical signal using a designed detection circuitry with output monitoring and data collection components [13,55]. An alternative strategy of ratiometric O_2_ sensing has been employed in recent studies to improve the readout, defined as the ratio of the O_2_-sensing dye readout signal or image to the non-O_2_ sensitive reference dye signal. Figure 1C shows an example of the ratiometric concept, where the standard deviation is proved to be half of the typical imaging intensity-based strategies [61]. In addition, few studies have used phosphorescence for on-chip O_2_ sensing, using the previously described luminescent dyes with longer excited-state lifetimes [62]. In these studies, the dyes were utilized for phosphorescence sensing of O_2_ with the same concept used in filmmaking strategies for luminescent methods [62,63,64].

One of the main ways to enhance the sensitivity and stability of the optical O_2_ measurement is via the fabrication techniques such as solvent-induced fluorophore impregnation (SIFI) method [41]. The method was recently developed which involves the impregnation of PtTFPP dye into the body of the cell culture chip without adding a dye-coating inside the chip. In this strategy, the challenges associated with patterning sensing layers in chambers, such as delamination and formation of cracks, were eliminated [41]. The application of optical fibers in O_2_ sensing has several advantages including the possibility for online and remote monitoring and miniaturization in O_2_ sensing devices [65,66]. Additionally, Yang and colleagues developed a capillary optical fiber (COF) with a ring-shaped waveguide used for O_2_ detection. They coated luminescent dyes on the inner surface of the waveguide in the COF for ratiometric O_2_ measurement with high sensitivity (high ratio of O_2_-sensitive dye signal over the control dye) and low response time (less than 7s). However, the main drawbacks of this method are the high cost and the need for special readout devices [67]. In many cases, O_2_ measurement needs to be performed simultaneously along other important parameters such as pH and cell metabolites to assess cell physiological condition. This can be carried out by several techniques such as using different dyes [43,58], micro-patterning the sensors in various layers of the chip [42], using optical fibers that have several excitation/emission wavelengths corresponding to O_2_ and pH [43] (Figure 1B).

#### 2.1.2. Microparticle-Based Sensors

Micro-sized materials such as microbeads and microspheres loaded with luminescent dyes have also been used in luminescent O_2_ sensing inside chip. This method benefits from simplicity of optical O_2_ detection and add up the advantages of microparticles such as higher surface area with higher interactive surface which enhance the sensitivity, protecting the dye from decay and also lower analyte diffusion distances that enable them to have a faster response. [17,41,67,69,70]. These microparticles are mostly made of polymers such as microparticles of polystyrene (PS), poly (dimethylsiloxane) (PDMS) and silica [65,70]. they have lower analyte diffusion distances that enable them to have a faster response [17,41,67,69,70].

Jiang et al. described a microfluidic-based method to make monodisperse PDMS microbeads containing PtTFPP dye for O_2_ sensing. After microbead characterization (150 μm diameter), they tested the efficiency of the O_2_ sensors by spreading them over the bottom of the microfluidic channel of the PMMA chip and exposing them to pure nitrogen (0% O_2_) and air (21% O_2_) flow. Similar to the conventional film layers, the phosphorescence effects of the microbeads were quenched by an increase in their O_2_ content (shown in Figure 1D) [68]. Their higher gas permeability, chemical inertness, nontoxicity, and biocompatibility have made them a more interesting choice for this application. In a relatively similar strategy, PS microbeads (3 µm diameter) with PtTPTBPF dyes were used for the 2D and 3D hydrogel-based cell culture chips which had a sensor spot to connect to a fiber optic and portable commercial FireStingO2 optical O_2_ m (Pyroscience, Aachen, Germany) [17].

A novel liver-on-chip with O_2_ monitoring has used tissue-embedded PS microparticles (50 μm diameter) loaded with ruthenium-phenanthroline-based phosphorescence dyes. The system was designed for the study of mitochondrial dysfunction using an organoid of HepG2/C3A cells [69]. Figure 1E shows that each microwell was first loaded with about 100,000 cells and 20 O_2_-sensing beads. The figure also illustrates the whole bioreactor (a) and its organoids-containing PDMS microwells (b), as well as fluorescent imaging of the organoid incubated overnight with O_2_-sensitive microbeads (c). The microbeads embedded in the tissue can also be used to assess the cell’s O_2_ uptake and metabolism rate in real-time measurement. The availability of a continuous O_2_ uptake measurement over 28 days was mainly due to the bioreactor design, in which the microbeads existed in the trapped cells and are involved in cell migration. This method also guaranteed the absence of any necrosis. The number and location of the microbeads during the experiment, therefore, were fixed over time. In another study with similar microbeads and concepts, O_2_ monitoring was carried out in a liver-on-chip to test different drugs [47].

Another team designed a microbioreactor for recapitulating intratumor O_2_ gradients to study the solid tumor microenvironment. The cellular metabolism and physical constraints of a cell layer (MCF-7 cells) between the two diffusion barriers were used in a tumor section-like culture method. The O_2_ gradient was mimicked without the need for using any O_2_-gradient making devices [45] (Figure 1F). In order to achieve the gradient, silica microparticles were loaded with an O_2_-sensitive luminescent dye (tris(4,7-diphenyl-1,10-phenanthroline) ruthenium(II) dichloride), which were glued to the pillar by a PDMS layer inside the microfluidic channels. The fluorescent imaging showed a uniform signal from the O_2_-sensitive microbeads, which could distinguish different degrees of hypoxia in real-time and perform spatially resolved measurements superior to conventional imaging-based systems in the OOC devices [45]. In another study, microbeads were used to simultaneously detect O_2_ and pH to study embryonic development inside a single zebrafish embryo culture system. Embryo culture microwells were composed of two sensing hydrogel (Poly(ethylene glycol) diacrylate (PEGDA)) layers. PS microspheres with Pt-porphyrin dyes were used to study the O_2_ consumption rate (OCR) while dextran microbeads loaded with a (2′,7′-bis-(2-carboxyethyl)-5-(and-6)-carboxyfluorescein (BCECF)) dye were applied for sensing the acid extrusion rate (AER). The O_2_-sensing system was reported to be efficient and capable of monitoring low OCRs of a single early embryo captured in a closed microwell [46].

#### 2.1.3. Nano-Enabled Sensors

The application of nanoparticles (NPs), nanostructures and nanocomposites in sensors has been of great interest over the past decade. This is mainly because of wide range of source materials, shapes and surface functionalization options which increase the flexibility of choosing the suitable nanoparticle for the sensing strategy of each study. The smaller size and/or pores of such materials increase their aspect ratio as well as their active surface, thereby improving their reactivity to a greater degree compared to micro-sized materials. The presence of functionalized groups for attachment to different surfaces, linkers and molecules on their surface expands their features and applications. The unique optical and plasmonic properties of NPs has great potential for enabling rapid and sensitive O_2_ optical sensing. Moreover, these NPs make the dyes more soluble and protect them from direct contact with the solutions which protect the dye from decay and also from potentially interfering molecules inside the chips and improve the selectivity and storage ability of the chip O_2_ sensor [71,72,73,74,75]. NPs based on polymers such as poly (methyl methacrylate) (PMMA), PS, poly(styrene-block-vinylpyrrolidone), poly(styrene-co-maleic anhydride), (poly(fluorene-alt-benzothiadiazole), are among the most commonly used types in microfluidic devices [65,70].

The application of NPs for O_2_ sensing in cells has been studied by the synthesis of PMMA NPs loaded with Pt(II) Octaethylporphine (PtOEP) dye and surface modification with Poly L-lysine (PLL), which makes the system biocompatible and easy to enter the living cells due to their positive charges. These NPs are efficient in sensing dissolved O_2_ (DO) concentrations as low as 0–43 ppm [71]. The NP-based O_2_ sensing has also been applied in microfluidic chips for bacterial growth monitoring. They benefited from PS NPs with platinum (II) 5, 10, 15, 20-meso-tetraphenyltetrabenzoporphyrin (PtTPTBP) dyes for rapid and simple monitoring of the metabolic activity of bacteria for 72 h [72]. In another study, Horka and colleagues used poly(styrene-block-vinylpyrrolidone) NPs with PtTPTBPF dye to monitor the metabolic activity of the bacteria via phosphorescence lifetime-based measurement strategies for O_2_ sensing inside a microfluidic droplet [49].

**Figure 2:**
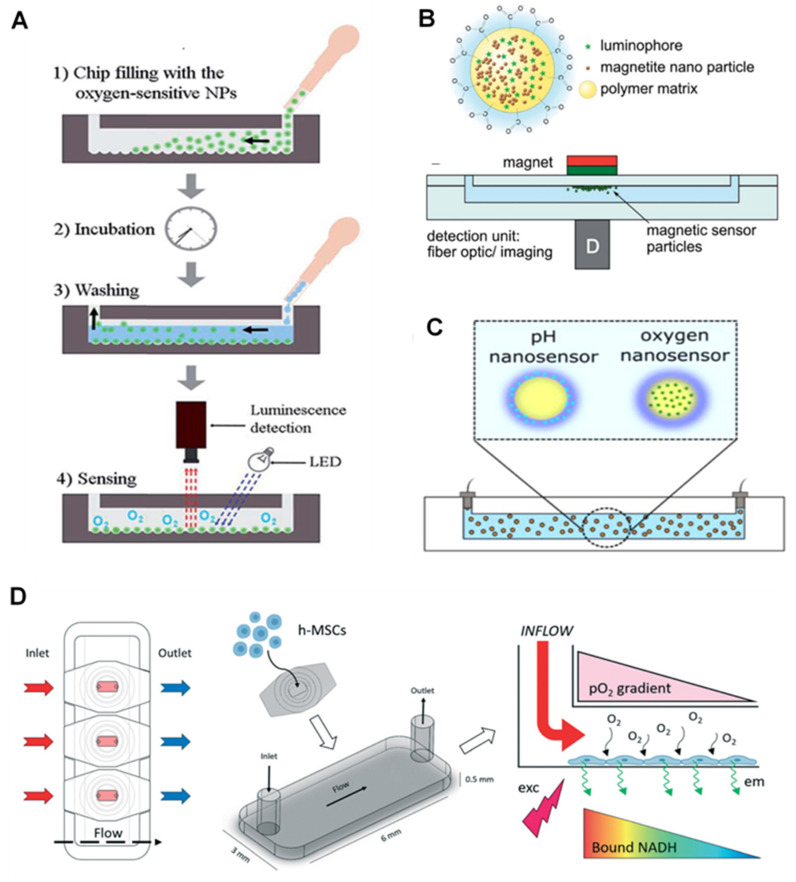
Application of nanoparticles for O_2_ sensors in on-chips. (**A**) Nanoparticles are introduced into the channels to attach to the microbeads inside the chip and form an O_2_-sensitive layer. Reproduced with permission [51], copyright 2015, The Royal Society of Chemistry. (**B**) Magnetic NPs with luminophore come together in a spot in the chip parallel to where the outside magnet is located. Reproduced with permission [76], copyright 2014, The Royal Society of Chemistry. (**C**) Sensitive dyes in core-shell NPs for simultaneous detection of O_2_ and pH. Reproduced with permission [76], copyright 2014, The Royal Society of Chemistry. (**D**) Chip-based stem cell culture to monitor O_2_ gradient via optical luminescent sensing layers. Reproduced with permission [48], copyright 2021, The Royal Society of Chemistry.

In the O_2_ sensors developed by Lasave and colleagues, polymer NPs (about 25 to 35 nm) were physically adsorbed on the silica microparticles at the bottom of the glass chip (Figure 2A). The comparison between glass chips with and without the microparticles as the NPs bed for O_2_ sensing showed that the chips with microparticles perform better compared to bare glass [51]. They showed that the sensors were stable with fast response and capable of using detection dyes (pH-sensitive dyes). The sensors could be used inside the microfluidic channels, even closed ones, by introducing the NPs. This method, however, is limited to glass chips (not plastic-based devices).

Magnetic NPs can also be used for chip-based O_2_ sensing. For instance, magnetic NPs and PtTFPP dyes (luminophore dyes) were merged into the poly(styrene-co-maleic anhydride) (PSMA) polymer to form a magnetic nano-complex. They were brought together via an external magnet over the chip’s sensing spot, while the imaging or optical fibers were used as the readout [51]. The sensing spots can measure not only the other O_2_ level but also the temperature and pH.

Core-shell poly(styrene-block-vinylpyrrolidone) magnetic NPs, with a PS core and hydrophilic polyvinylpyrrolidone shell, were used for simultaneous detection of O_2_ and pH. PtTPTBPF as an O_2_-sensitive dye is incorporated in the core, where the pH indicator and the reference dye are applied on the shell (Figure 2B,C) [76]. Based on the reported results, a mixture of NPs loaded with O_2_, and pH dyes performed better than those containing both dyes. This strategy allows simultaneous detection of the two parameters and is easy, reproducible, and stable in different circumstances. Scientists also introduced a perivascular niche-on-a-chip device, in which the intracellular levels of O_2_ were monitored in human mesenchymal stem cells (h-MSCs) using polyfluorene NPs (poly(fluorene-alt-benzothiadiazole)) covalently conjugated with dyes (platinum(II) meso-bis(pentafluorophenyl)bis(4-bromophenyl)porphyrin (PtTFPPBr2)). They used NAD(P)H fluorescence signal as a reference in a fluorescence lifetime imaging microscopy (FLIM) system (Figure 2D). The applied NPs are reported to have a high-intensity fluorescent signal with high cell uptake levels, resulting in the chip-based real-time assay of intracellular O_2_ levels. These NPs can also be easily applied to closed chips [48].

#### 2.1.4. Commercial Probes and Portable Devices

**Figure 3:**
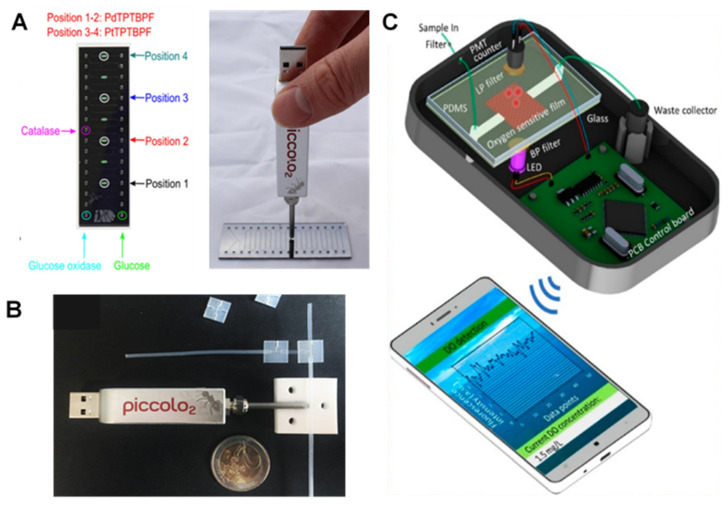
Commercial and portable optical readout systems for on-chip O_2_ monitoring. (**A**) A chip (left) designed for commercial optical readout devices (right). Reproduced with permission [78], copyright 2016, Elsevier. (**B**) chip-based bacteria study with O_2_ measurement through the wall of tubing using a commercial readout device. Reproduced with permission [49], copyright 2016, American Chemical Society. (**C**) A portable handheld photodetector device connected to a mobile app for chip-based O_2_ monitoring. Reproduced with permission [80], copyright 2021, MDPI.

Commercial and/or portable modules have been used for on-chip monitoring of O_2_ levels in embryo, cell and tissue cultures [17,77]. While the benefits of portable O_2_ sensing in microfluidic devices are well known, in OOCs, portability can bring additional features such as compactness and miniaturization of the sensing module, an automated, simple working platform with their own software, and multiplex detection of factors such as pH, CO_2_, and temperature besides O_2_. In this regard, few companies have introduced commercial portable Universal Serial Bus (USB)-based optical readout systems such as FireStingO2 (PyroScience, Aachen, Germany) [17,77], PiccolO2 device (PyroScience, Germany) [49,50] or portable detection modules such as VisiSens device (PreSens, Regensburg, Germany) [77]. These optical readout modules can be mounted on (Figure 3A) or connected to (Figure 3B) the sensing spot on channel/chamber or implemented in a modulated box (Figure 3C).

For Example, Ehgartner et al. introduced sensor spots integrated with silicon-glass microreactors connected with commercial optical readout devices [78,79]. Figure 3A represents the chip and its sensing spots (left) as well as the commercial picolo2 readout with a USB port (right) which can be connected to the computer for further analysis. The chip was designed to measure the O_2_ level and to monitor the enzymatic activity of D-amino acid oxidase and glucose oxidase as model enzymes. Seven cell culture chambers and four O_2_ sensing spots were read by a simple USB O_2_ m (Piccolo2) connected to an optic fiber or by a four-channel optical O_2_ m (FireStingO2). The chip was compatible with two famous luminescent O_2_-sensitive dyes, with two for PdTPTBPF and the other two for PtTPTBPF. The method, therefore, was concluded to be accurate with an automated and inexpensive production line and compatible with commercial readout devices. In another example, but for monitoring the metabolism of bacteria in droplets, Horka et al., also used Piccolo2 device enabling the non-invasive contactless measurement through the wall of a tubing as it is shown in the Figure 3B [49].

Zhu et al. introduced a microbioreactor for monitoring metabolic activities of zebrafish embryos with a sensor foil integrated with O_2_ monitoring using a Fluorescence Ratiometric Imaging (FRIM) system [77]. An O_2_-sensing foil was integrated into an embryo trapping chamber in a PMMA chip. A portable USB-embedded commercial ViviSens detector was used for fluorescent imaging. A calibration curve was plotted before the embryo culturing for chip-based O_2_ measurements. The method was proved to provide a real-time and non-invasive measurement for the embryos. Other advantages were the possibility to use commercial O_2_ sensing devices and software, such as a small integrating and portable detector with USB port, and the capability of measuring O_2_ gradient.

In another development, Wang and colleagues developed a portable detector for O_2_ detection using a PDMS chip and a PtTFPP film [79,80]. The detector (Figure 3C), comprised of a photomultiplier (PMT) counter (with a Charge Coupled Device (CCD) camera and an imaging spectrograph) connected to a smart-phone application and was able to detect DO levels with an LOD of 0.01 mg/L (0.37 µM) and short response time of 22 s. The simplicity, low-cost production, handling of the device, high sensitivity, short response time and portability are among important features which make this detector a desirable device for on-chip O_2_ sensing, especially in medical applications.

### 2.2. Electrochemical Methods

Electrochemical (EC) sensing is a common technique to measure low concentrations of DO, often in microfluidic applications, with high sensitivity [81,82]. Being introduced over two decades ago, it is believed that certain shortcomings of optical measurement techniques are resolved by using EC sensing, such as the need for time-lapse bright field and fluorescence microscopy in combination with various staining techniques as well as a collection of supernatants and cellular samples traditionally used in the OCCs. However, the direct integration of EC-based DO sensors in OOC systems is not common because of the complexity, multi-step and high-temperature fabrication process and risk of material incompatibility, all of which increase the complexity of the whole system as well as the overall probability of failure [12]. In the following subsections, we review several innovations to overcome such difficulties in EC sensors and categorized into five main innovation groups: (i) novel configurations in Clark-type sensors; (ii) novel materials (e.g., polymers, O_2_ scavenging and passivation materials); (iii) nano-enabled electrochemical sensors; (iv) novel designs and fabrication techniques; and (v) commercial and portable electrochemical readouts. Table 3 summarizes the components, advantages and LOD of the selected EC sensors for the measurement of DO.

#### 2.2.1. Novel Configurations in Clark-Type Sensors

The Clark-type configuration has transcended the main limitations of the optical sensors, such as high cost and complicated fabrication process. The system normally consists of an electrode covered with a thin layer of electrolyte solution and a thin polymer membrane enabling selective permeation of O_2_ to the immersed electrode. The membrane also separates the electrolyte from the specimen, preventing cross-sensitivity and resulting in a short turnaround time. However, the need for mechanical agitation, circulation of the sample or immersion of the sensor in the sample (which requires large amounts of sample and reagents) are the main drawbacks of such sensors [81,97]. Apart from the high risk of mechanical failure in the thin gas permeable membrane, such sensors are not compatible with continuous monitoring, automation and high-throughput measurements [98].

Reducing the size of the sensor and/or limiting the measurement time is necessary to reduce O_2_ consumption by the EC sensor and to prevent possible endangerment of the biological sample. This is important for the measurement of the kinetics of O_2_ variation in samples and for monitoring the cell growth. Therefore, using a micro total analysis system (μTAS) has overcome some of these challenges. O_2_-plasma bonding was one of the first methods used in miniaturized Clark O_2_ sensors [83]. Unlike the field-assisted bonding technique, which requires high electric voltage (kV) and temperature (250 °C) to combine the glass and silicon substrates, this simple and low-temperature fabrication process enabled the integration of several semiconductor elements and polymer-material structures in an ordinary laboratory environment [99]. The use of thick-film technology with the SU8 photoresist and extremely thin O_2_ separation membrane resulted in a low response time (6.8 s). However, the sensor was not suitable for long-term measurements.

In a more recent attempt, a low temperature co-fired ceramic (LTCC)-based microfluidic Clark-type O_2_ sensor was used for real-time monitoring of localized DO [84]. The advantages outlined for the LTCC materials include hermeticity and mechanical durability, high scalable prototyping/manufacturing, and the ability to directly integrate both electronic and microfluidic with a compact 3D package [100,101,102]. The EC sensor consisted of a solid proton conductive electrolyte that facilitated the fabrication process and improved shelf life. The solid-state proton conductive matrix (PCM) membrane (Nafion 117 membrane) was used as a support to lower the risk of mechanical failure of the PDMS O_2_ permeable membrane (OPM). Also, the microfabricated electrodes and continuous flow of the sample through the microchannel could reduce the O_2_ depletion risk.

**Figure 4:**
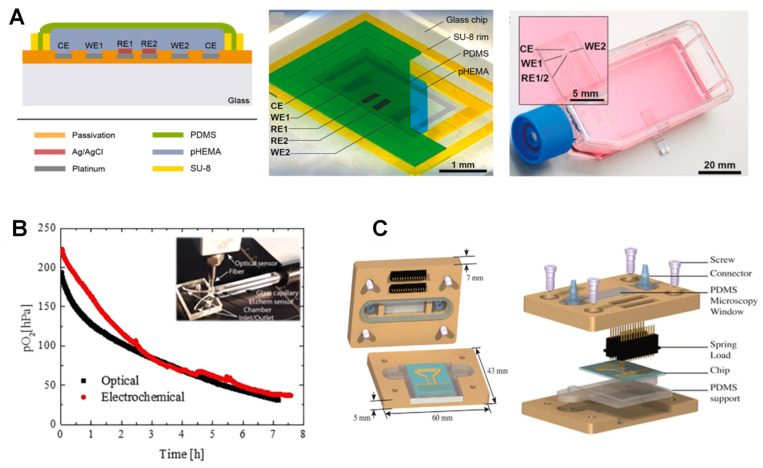
Electrochemical sensors for on-chip O_2_ measurement. (**A**) cross-section and top view of the Clark-type O_2_ monitoring microsensor via chronoamperometric sensing protocols in cell culture and organ-on-chip systems. Reproduced with permission [85], copyright 2020, Elsevier. (**B**) microfluidic cell culture chip with intra-channel parallel electrochemical and optical O_2_ measurement using commercial optical and electrochemical readout devices showing agreement between the results (red and black curves). Reproduced with permission [105], copyright 2019, American Chemical Society. (**C**) Components and cross-section of the microfluidic chip with electrochemical detection electrode arrays for a distinct set of three-electrode for O_2_ and the electrode array for Na^+^, K^+^ and pH measurement. Reproduced with permission [106], copyright 2015, Elsevier.

Clark-type O_2_ microsensors also promise zero analyte consumption if the feedback mode (Ross principle) is successfully implemented [103]. According to this principle, a suitable sensor configuration allows for the compensation of O_2_ consumption at the working electrode (WE) through evolving O_2_ at the counter electrode (CE) while maintaining the sensor pH. This is confirmed by showing O_2_ formation as the only oxidation reaction at the CE. Another example is a microsensor with Pt as WE was fabricated on a glass chip using thin film technology. The use of a poly-2-hydroxyethyl methacrylate (pHEMA) hydrogel layer containing buffer solution as an electrolyte and PDMS as a gas-permeable membrane showed promising results in monitoring cell respiration in cultures (Figure 4A) [85]. Thanks to the dried-out hydrogel layer, the microsensors could be stored dry and activated by immersion into aqueous analytes. As a result, the measurement in the gas atmosphere, even with ambient humidity, was possible only for less than one hour. Chronoamperometric protocols to renew the electrode surface through the formation of PtO and subsequent removal before O_2_ measurement was applied to enable long-term stability, for more than one week, without any need for recalibration.

In order to simplify and speed up the prototyping process, a confined microfluidic cell culture system was developed using a microscopic indium tin oxide (ITO) slide with planar Pt sensors at the bottom of a plate for the measurement of acidification, O_2_ consumption, and cell adhesion [104]. The structure of the slide, along with the gas permeability of the PDMS lid, ensured 100% air saturation in the culture medium, desirable for the cells but responsible for possible bubble formation. Using an ultrashort pulse laser, the fabrication process of such amperometric Clark O_2_ sensors took about three minutes per chip. This not only improved the precision of the chip but also prevented possible exposure to toxic chemicals or adverse effects in the cell culture.

In a clinical application of a multi-analyte OOC, dynamic measurement of DO was performed in low volumes of urine using Clark-type microsensors with a high sensitivity of 3.60 ± 0.2 nA mg L^−1^ [86]. The multi-sensor platform was composed of three different polymeric layers: a 127 mm thick polyimide film (Kapton^®^ 500HN, DuPont Co., Wilmington, DE, USA incorporating the gold microelectrode array, a 175 mm structured Polyethylene terephthalate (PET)-two-side adhesive sheet (AR8939) defining the microfluidic manifold, and a 188 mm cyclo-olefin polymer (COP) film (Zeonex ZF14-188, purchased from Ibidi, Gräfelfing, Germany) as the cover. The use of flexible polyimide Kapton^®^, besides its thermal and mechanical stability, high chemical resistance, and low dielectric constants, ensured the platform was cost beneficial.

#### 2.2.2. Novel Materials: Polymers, O_2_ Scavenging and Passivation Materials

As mentioned in the previous section, polymer membranes are an important part of EC-based O_2_ sensors. This is because O_2_ is diffused to the reaction chamber, where it is consumed, while biological cells and other organics are prevented from passing through. PDMS is the most common polymer used to construct the microfluidic channels and the sensor support because of its characteristics, such as biocompatibility, gas permeability, mechanical flexibility, and optical transparency.

In an integrated microelectrochemical reactor, an EC sensor with incorporated Pt interdigitated array (IDA) as WE, a Pt CE, and Ag pseudo-reference electrodes (RE) were used. The WE was fully immersed in a liquid electrolyte confined in the channels [107]. The O_2_ reduction reaction (ORR) was used in this platform for both pH tuning and fluid actuating reactions. In this regard, a microelectrochemical cell design inherent to membrane-covered Clark-type sensors was used. Unlike common polymers that are less permeable to O_2_ than the electrolyte, a highly permeable PDMS membrane, capable of supporting significant current densities, was used. This simple change enabled remarkable pH gradients capable of initiating and/or sustaining marked fluid displacements within the microfluidic channel system with low applied potentials (0.1 V) and no need for additional electrochemically active components. Due to the high O_2_ permeability of PDMS, the microfluidic cell supported significantly higher current densities in the ORR compared to those measured in conventional (quiescent) EC cells with similar electrode areas. This results in achieving more stable ORR currents. Another advantage of this system is the absence of bubbles. Using membranes thinner than 0.1 mm allows for a higher diffusion rate with limited ORR current densities. The significant O_2_ mass transfer in the PDMS structures of the microfluidic cell helped generate transient chronoamperometric waveforms while sustaining the steady-state mass transfer limited the ORR current densities.

As another example, a multi-sensor glass chip with a PDMS imprinted microfluidic channel grid was developed to characterize cellular behavior [87]. The microfluidic manifold was fabricated based on the Haversian bone-canal system to ensure a homogeneous flow. They resembled a Clark-type amperometric sensor without any O_2_-selective membrane. The glass-wafer technology ensured the microscopic observability of the on-chip cell culture along with online monitoring of the physiological parameters. On the other hand, glass is less costly compared with silicon-sensor technology [88,108,109,110]. Circular 78.5 μm^2^ Pt electrodes, as O_2_ sensors, led to a sensitivity of 100 pA per each 1% increase in saturated air (21% O_2_). The sensor worked for 80h during cell culture, and the fluctuations noted in the current rates were possibly due to large gas bubble formations.

Although PDMS is the most common polymer used in this regard, other polymers have also been tested. PTFE has higher permeability and faster O_2_ diffusion rates compared to PDMS, which results in O_2_ depletion in the channel [111]. Adjusting the electrolyte flow rate and the channel length can ensure the presence of sufficient O_2_ in the biomass chamber and thus the reliability of the results. However, PDMS-based respirometers allow shorter analysis times, especially when thicker membranes are needed or the sample is rich in organic matter. Using gas permeable membranes such as PDMS alone has limitations such as loss of vapor which changes the fluid composition of the microfluidic cultivation chambers and may lead to reoxygenation from the ambient air. Other materials benefit from diffusion and mixing deoxygenated fluids, chemicals, electrolytic, photocatalytic, and biological O_2_ depleting liquids such as sodium sulphite and pyrogallol [112]. These materials, however, can interfere with the cellular processes and metabolic pathways. Using an external gas supply with nitrogen to equilibrate the O_2_ concentrations is another alternative, even though it is less accurate [113].

Functional microfluidic materials with intrinsic O_2_ scavenging properties allow for long-term cell cultivation under reduced O_2_ concentrations. These are cheap substitutes for bulky and expensive hypoxic chambers. Adjusting the temperature and curing time during the process of fabrication and altering the microfluidic layout and the surface area-to-volume aspect ratios of the channels, as well as modifying the flow rates during cell cultivation, can help fine-tune the O_2_ scavenging rates and address specific biological issues. In an example of a miniaturized OOC with embedded O_2_ sensors, an off-stoichiometric thiol-one epoxy polymer was integrated into a functional biochip to efficiently remove DO to below 1.0 hPa. In a clinical application, this system was used to study acute ischemic stroke using a murine blood−brain barrier (BBB) model (Figure 4B) [105]. In this system, anaerobe bacteria were cultivated under ambient air until efficient germination of pathogens was achieved. They compared two parallel O_2_-sensing methods: an opto-chemical O_2_-sensing based on luminescence lifetime measurement using an O_2_ indicator luminescent dye (PtTPTBPF) and an EC sensor directly detecting DO levels by oxidation at the electrode surface measured by a commercial readout OX-NP Clark-type sensor (Unisense, Denmark). Both sensors had similar time-dependent O_2_ depletion profiles, resulting in O_2_ tension of 30.76 and 37.58 hPa after 7 h, respectively. They showed similar DO removal from about 200 to 30 hPa within 7 h in a 25 μL microchannel volume. O_2_-sensitive microbeads, however, were selected as the best options because of their smaller footprint and ease of integration within the microchannels.

Passivation materials are also used to not only define the electrode surface but also ensure their long-term EC stability. In an attempt to develop a low-cost, easy-to-use, reproducible and portable microfluidic device, a specific B33 glass substrate was used [91]. The idea was to initiate and monitor microalgae photosynthesis activity while electrochemically assessing the microalgal O_2_ production rate during artificial night/day cycles. The ElecCell (Electrochemical microcell) technological platform, with fully integrated EC microcells (titanium/platinum (Ti/Pt) as the working microelectrode, the counter and the sub-structure of the pseudo-reference) was developed using double physical vapor deposition (PVD). A silicon nitride (SiN_x_) wafer-level was deposited for passivation using low-temperature PVD. The microfluidic system was fabricated through laminating dry films to overcome the need for any micropumps or other fluid actuation devices.

In another scalable microfluidic platform developed for continuous monitoring of biofilm proliferation and activity under shear stress conditions, a double layer of PVD deposited silicon oxide and silicon nitride (Si_3_N_4_) was applied for passivation purposes (Figure 4C) [106]. The combination of two interdigitated microelectrodes (IDuEs) and punctual electrodes helped with the measurement of DO, K^+^, Na^+^ and pH. The optimized IDuEs permitted sensitive and reliable label-free monitoring of *Staphylococcus aureus* V329 growth. The four-electrode system eliminated possible electrode-electrolyte effects, improving sensitivity and providing morphological and structural information necessary for assessing the bacterial growth on the electrode. A combination of polycarbonate and PDMS was used to fabricate the upper and lower lids, respectively. The novel platform could thus be integrated into multiple environments, allowing simultaneous optical microscopy and impedance spectroscopy measurements.

#### 2.2.3. Nano-Enabled Electrochemical Sensors

Nano-enabled sensors are those types use nanomaterials or nanolayers of materials in order to enhance the sensitivity of the oxygen measurement and electrical and mechanical properties of the used materials. Nafion is a permeable membrane that enhances the electrodes’ shelf life and their sensitivity due to its anti-fouling properties. In an example of miniaturized cell-based biochips for toxicological analysis during cell growth and development, EC amperometric sensors were integrated at the inlet and outlet microchannels of a PDMS cell chamber to perform real-time continuous glucose and O_2_ monitoring [89]. Two arrays of thin-film gold microelectrodes (0.16 and 0.016 mm^2^ for glucose and O_2_, respectively) were incorporated in a biocompatible PDMS microfluidic cell culture device. Nafion-modified O_2_ sensors could measure O_2_ concentrations from 237 to 50 mmol L^−1^ with a sensitivity of 0.50 ± 0.05 nA mol^−1^O_2_ L^−1^). The integrated Au/Nafion electrodes were concluded to be suitable for DO monitoring in dynamic flow conditions with high stability and short response time. The flow rate, however, needed to be controlled due to the high O_2_ permeation through the PDMS.

A lab-on-a-tube (LoT) integrated with spirally-rolled pressure, temperature, O_2_ and glucose microsensors was developed for multimodal real-time neuromonitoring as well as draining CSF in patients with traumatic brain injury [90]. The approach was reported to be less invasive and cheaper compared with traditional techniques. The temperature and DO sensors helped adjust the output, reducing errors commonly seen in in-vivo biosensors. DO sensors, the focus of the current review were fabricated on 25 mm thick Kapton film patterned with Ti/Ir/Au and finally parylene layers. The Au layer on the reference electrode was selectively etched, and iridium oxide (IrO_x_), a promising material for in-vivo reference electrodes, was grown anodically in a 0.7 M Na_2_HPO_4_ solution. Nafion and silicone membranes were deposited as a polymer electrolyte and O_2_ permeable layer, respectively. With a sensitivity of 12.89 nA mmHg^−1^, DO sensors could continuously monitor parentage of O_2_ of 152 mmHg (air-saturated) and 38 mmHg (5% O_2_) for at least five days with less than 9% sensitivity error.

#### 2.2.4. Novel Designs and Fabrication Techniques

The use of an ultra-microelectrode array (UMEA) for ultra-short (<5 ms) measurements is another novel technique to monitor DO concentrations in any solution [92]. As an example, the sensor consisted of Pt electrodes recessed in a glass substrate with oxide-nitride-oxide (ONO) as the insulating material. Such sensor was reported to have a high sensitivity of 0.49 nAs−0.5/mg/L with a drastically (about 10 times) low OCR, valuable for in situ assessment of the microtissues’ respiratory activity. One of the main limiting factors for the lifetime application of such sensors is the need for repeated recalibration. This drawback has been addressed by arranging the cell cultures and sensors in a multi-well system in a way that they come in contact with each other at certain times and then separated at others such that the measurement would have no impact on the cell growth [93]. The bottom side of the cultivation wells, made of 0.1–0.3 mm thick Al_2_O_3_ membrane with interconnecting pores (200–300 nm), was connected to the sensor area using a special coupling mechanism. The planar multi-sensor chip was made of screen-printed electrodes (SPEs) on the Al_2_O_3_ substrate, with those dedicated to O_2_ measurement covered with pHEMA and PUR membranes using dispensing technology. The gap between the two modules enabled the repeated flow of the analyte-containing liquid through a biocompatible nano-porous sterile membrane, resulting in continuous pH, glucose and O_2_ measurement during the cultivation phase. The system could be reutilized after sterilization with gamma-rays.

Inkjet printing (IJP) is a novel technique used for sensor fabrication. The main advantage of this technique is its compatibility with delicate substrates that cannot withstand high temperatures due to the possibility of drop-on-demand material deposition. Moreover, the direct writing approach without masks reduces the overall cost and fabrication time considerably and facilitates iterative design changes. Multiple sensors are integrated into an extremely thin, porous, and delicate membrane inside the OOC in such applications. The DO sensors are printed along the microfluidic channel allowing local online monitoring of O_2_ concentrations. A primer biocompatible dielectric layer (SU-8) is commonly used to seal the porosity of the membrane at defined areas, build a uniform deposition of conductive inks, accurate definition of the electrode area, and prevent possible short circuits [114]. In one example, DO sensors were fabricated using IJP in ExoLiver, a liver-on-a-chip device for real-time monitoring of the cell culture, O_2_ gradient, and OCR [94]. ExoLiver, a modular bioreactor consisting of two plates separated by a porous membrane, mimics the liver sinusoid system. The 300 μm DO sensors had a negligible O_2_ consumption of about 2.94 × 10 ^− 8^ mg s^−1^ per sensor, therefore not affecting the viability of the cell culture. They were calibrated by polarization at −650 mV, an optimal reduction potential value for determining DO concentrations without interfering with the electro-active medium. The sensor had a sensitivity of 28 ± 1 nA L mg^−1^ (range, 0–9 mg L^−1^) with a turnaround time of the 60s. A max of 17.5% gradient measured between the system inflow and the outflow could be due to the metabolic activity of the sinusoidal hepatocytes and cell consumption along with the bioreactor’s lower plate. This is of great value as the O_2_ gradient has a critical regulatory role, as it is directly linked with the metabolic zonation, morphology and xenobiotic transformation in the hepatocytes.

In another clinical application, the multi-analyte microphysiometer (MAMP), a modified Cytosensor Microphysiometer combined with additional amperometric glucose, lactate, and O_2_ sensors, enabled real-time measurement of changes caused by the metabolism of cells immobilized in a microfluidic chamber [115]. The unique combination allowed for the monitoring of both aerobic and anaerobic respiration. The main benefits of the planar SPEs include simple fabrication, versatility, high reproducibility, and low cost, all resulting in a simplified microfluidic chamber. A Dimatix materials inkjet printer was used to deposit the enzyme and polymer films on Pt SPEs to fabricate the glucose, lactate, and O_2_ sensors. This process guaranteed the homogenous coating of the electrode surface and its reproducibility. Low concentrations of BSA-PB solution instead of BSA also reduced the risk of bubble formation. A newer generation of MAMP was later developed consisting of five modifiable Pt electrodes along with an Ag/AgCl quasi-reference, designed to measure glucose, lactate, O_2_, and pH simultaneously in a single microfluidic channel [116]. Reproducible surface modification was again performed using IJP. The O_2_ sensor was modified with 2.5% Nafion. The highly adaptable system could act as a microphysiometry platform with a lifetime of up to 6 weeks.

Optical transparency of the OOC chip is obligatory for ongoing monitoring of cells using phase-contrast microscopy. This is while most of the existing microphysiometers, i.e., the Cytosensor^®^ and Bionas Discovery™ 2500 system, are silicon chips [117,118]. To overcome these shortcomings, an optically transparent multi-parametric microphysiometer was developed for continuous dynamic measurement of pH, O_2_, lactate and glucose in T98G human tumor cell cultures [88]. The biocompatible glass-based chip was composed of EC microsensors fabricated using a hybrid thin film and laminate technology. Microfluidics enabled controlled medium exchange and substance exposure to low volumes (<10 μL) with low flow rates (2 μL/min), reducing shear stress on the cells. The sensors were located upstream in the inlet channel (control), inside the cell cultivation area and downstream in the outlet channel. In order to separate the biosensors from the cell culture area, protect the cells from hydrogen peroxide exposure and prevent O_2_ depletion during cultivation, the fluidic channels and electrodes were fabricated using a permanent epoxy resist and were partly covered with a laminated polyimide film. The amperometric reduction of DO at the thin-film Pt-based circular electrodes of the O_2_ sensors resulted in a sensitivity of −0.735 μA μM^−1^ cm^−2^ (±0.013). The sensor, however, showed a 10% decrease in sensitivity when first entered into the cell culture medium, possibly due to protein adsorption on the electrode. The O_2_ sensors were reportedly stable for the long-term with linear behavior and negligible O_2_ consumption (<3%).

#### 2.2.5. Commercial and Portable Electrochemical Readouts

Commercial products have been used in EC methods for the measurement of O_2_ inside the chips. For example, the liver-on-a-chip device developed by Moya and colleagues in 2018 [94] and the blood–brain barrier (BBB) model developed by Sticker and colleagues in 2019 [105] have used the OX-NP Clark-type commercial EC readout device by Unisense Co., Denmark. A modular portable method was developed for cell culture monitoring [93]. The Bionas Analysis System 2500 (Bionas GmbH, Warnemünde, Germany, www.bionas.de, accessed on 20 October 2021) was used for in-vitro, non-invasive and parallel measurement of metabolic parameters of respiration, acidification and cell adhesion in time-frames ranging from minutes to days [119]. The Clark-type sensors detected O_2_ mediated current (charge transfer rate) with a sensitivity of 0.12 pA·s^−1^ ± 0.21. The use of the Koester coating protocol helped ensure comparable conditions for cell growth on different surfaces [120]. The inhomogeneity in cell distribution and growth over the entire chip surface, in practice, resulted in variations in values measured by the same sensors of the chip.

Another miniaturized EC respirometer monitored DO concentrations in water samples semi-continuously, unlike traditional expensive biochemical O_2_ demand (BOD) methods [121]. The device was composed of a double-flow cell, reaction chamber, housing the sample and microbial mixture, and an electrolyte chamber, separated by a thin membrane from the bioreactor to provide sufficient flexibility to perform measurements in a wide range of organic matter concentrations. In another attempt, a novel encapsulation design and a membrane barrier material, the Intelligent Mobile Lab for In-Vitro Diagnostics (IMOLA-IVD), was used for cellular microphysiometry [96]. Extracellular acidification (EAR) and O_2_ uptake (OUR) rates were passively monitored using a modular, label-free EC platform. A three-electrode membrane-free Clark cell was used to measure the DO levels. Above and under the 3D multicellular spheroids, the integrated layers allowed fluidic contact between the spheroids in microwells and the BioChip sensors while preventing any washout from medium perfusion.

The 3D cellular models mimicked the native environment of the tissue; however, their increased size and geometry, as well as limited access to O_2_ and nutrients due to restricted diffusion into the scaffolds, were the main limiting factors [122]. Multicellular spheroids incorporated with OOC platforms are a promising solution. EC microsensors with a small cross-section can be inserted directly into a microtiter plate well containing a single spheroid, microtissue or organoid. Due to their high sensitivity, excellent selectivity and defined zero-point, such EC sensors can measure small changes in O_2_ concentrations in the microtiter plate caused by the spheroid metabolism [95].

## 3. Conclusions and Future Perspectives

In this review, we discussed recent advancements of oxygen sensors in on-chip systems and categorized them in two main groups: optical and electrochemical methods. We also discussed recent research and novelties in each section with schematic Figures and an overview Tables. The optical methods are reported to be more sensitive, easier to operate and cheaper compared to the EC methods. In most cases, they do not consume O_2_ during the process. In addition, they are compatible with commercially available luminescent dyes and optical readout devices, even fluorescent microscope which is convenient and available in most cell and tissue process centers. These sensors can be used for contactless monitoring by adding a sensing spot outside the chip readout or optical fiber, making the handling and sterilizing of the chip simpler. In addition, they do not need recalibration or experience decay over time. Compared with EC methods, therefore, these techniques are more commonly used in OOC applications. This is especially important for low concentrations of the sample when the stability and reusability of the sensor and its remaining intact are crucial. On the other hand, the EC methods have a shorter response time, and in most cases, a higher sensitivity than the previous group. In most cases, they can be used as label-free sensors, which again reduces the cost of sensor fabrication compared with optical ones. However, their integration in the chip is relatively expensive, complicated, and sometimes requires special relatively expensive instruments (Potentiostat/Galvanostat) and skilled operators.

In the future, the use of novel materials, fabrication techniques, and chemical/physical surface modifications can help facilitate the fabrication steps, reducing the price and required specialty, making the chip-integrated O_2_ sensors more affordable. Although there are few examples of micro- and nanomaterials used for the chip-based O_2_ sensors of either type, the field is rapidly progressing. It is expected that a variety of materials with various properties to help with O_2_ sensing will be used for these sensors in the near future to improve their sensitivity. This is mainly because these materials are believed to increase the active surface area, enhance the stability of the surface and increase the glow of the dyes. For instance, graphene and quantum dots can be considered as potential materials due to their exceptional optical and electrochemical properties. Micro- and nanostructures of novel metal oxides are other examples of such materials because of their catalytic activity. In addition, quantum dots (QDs) are photostable and their excitement spectra are broad, while their emission spectra are size-tunable, which makes them suitable for optical sensors. Further, using a smartphone to quantify the emitted signal can significantly simplify the measurement process. Artificial intelligence and innovations in image and signal processing help enhance the sensitivity and specificity of O_2_ sensors. Additionally, using 3D printing to manufacture the sensors can result in flexible, rapid and low-cost designs and integration of sensors in OOCs, and improve the sensitivity of the O_2_ sensors inside the chips. Three-dimensional printing techniques can also be used to build customizable optical holders and modules that can be integrated with mobile phones or other portable detectors.

## Figures and Tables

**Table 1:** Comparison of chip-based electrochemical and optical O_2_ sensors.

Method	Advantages	Limitations
Optical	Precise, sensitive and selective Easy to miniaturize Nano and microparticles for dye protecting Non-invasive, and contact-free Easy to use and operate Commercial dyes and read-out devices Simple handling and sterilizing of the chip Less need to recalibrate Multiplex measurement in different chip spots Simultaneously measure pH and metabolites	Complicated integration into the chip Possible dyes bleaching Sometimes needs microscope
Electrochemical	Precise, sensitive and selective Easily miniaturized/implemented inside chips Nano and microparticles for dye protecting Possible use of commercial read-out devices Short response time High sensitivity Label-free Several electrodes Several surface modifications Several designs and polymers can be used easily	Invasive and consume oxygen Expensive integration in the chip Requires special instrument and skilled operators

**Table 2:** Characteristics of chip-based optical O_2_ sensors.

Optical O_2_ Sensor	Application	Dye	Advantages	References
Polystyrene chip, pore network structure, used solvent-induced fluorophore impregnation (SIFI) method for dye layer	Cell	PtTFPP	Enhanced sensitivity and stability, non-invasive, can be used for gas and dissolved O_2_	[41]
PDMS chip with glass layer coverage, applied oxygen gradient	Liver	PtOEP	Wide dynamic range, continuous measurement, non-invasive, worked in different flow rates	[42]
Cyclic olefin copolymer-based chip	Lung	PtTPTBPF	Simultaneous O_2_ and pH, stop/flow measurements, long term stability (10 days), non-invasive	[43]
PDMS chip, applied oxygen gradient	Cancer	PtOEPK	Photostable, reusable, non-invasive	[44]
PDMS chip, silica microparticles	Cancer	Ru(dpp)	Simple fabrication and handling, real-time, spatially-resolved measurements, low photobleaching, High sensitivity	[45]
PMMA chip, polystyrene microspheres	Embryo study	Pt-porphyrin	Simultaneous O_2_ and pH, long-term measurement, highly sensitive for single embryo analysis	[46]
PDMS chip, polystyrene microbeads	Liver	ruthenium-phenanthroline (RuP)	Every 15 min for 28 days measurement, without a decrease in signal loss and toxicity, simultaneous glucose and lactate measurements	[47]
Glass chip, nanoparticle probes	Stem cell	PtTFPPBr2	Highly sensitive, real-time, label-free, high-intensity fluorescence emission, cell permeability	[48]
Teflon fluorinated ethylene propylene (FEP) tubing, poly(styrene-block-vinylpyrrolidone) nanobeads	Bacteria	PtTPTBPF	Minimized background fluorescence, simultaneous measurement, highly soluble and disperse nanobeads, prevents any interferences from biomolecules, short response times, no dye leaching, and long storage periods	[49]
Silicon/glass chip, core−shell nanosensors (poly(styrene-blockvinylpyrrolidone)	Fibroblast cell	PtTPTBPF	Simultaneous O_2_ and pH, contactless and inexpensive read-out, high ionic strength, highly stable, online monitoring	[50]
Glass chip, polymeric nanoparticles	Cell	Pt(II) benzoporphyrin	Highly stable at different pH, ultrafast response (less than 0.2 s), no leaching, repeatable	[51]

**Table 3:** Characteristics of chip-based electrochemical O_2_ sensors.

EC-Based O_2_ Sensor	LOD	Advantages	References
PDMS-container structure, and the glass substrate	105 cells/mL	Short response time (6.9 s)	[83]
Low-temperature co-fired ceramic (LTCC) in an improved Clark-type DO sensor	Up to 8.1 mg/L	easy fabrication, flexible configuration, short response time (10.9 s), real-time detection	[84]
pHEMA hydrogel layer with electrolyte and PDMS as gas-permeable membrane	0.121 μA cm^−2^ μM^−1^	zero analyte consumption, 1-point calibration, long-term stability	[85]
PPy as the internal contact layer between polymeric sensitive membrane and gold	0.11 ± 0.02 mg L^−1^	Low cost, good performance and long-term potential stability	[86]
Multi-sensor glass-chip with a PDMS imprinted microfluidic channel grid	100 pA per each 1% O_2_	Transparent for microscopic observation, cheap, high sensitivity	[87]
Biocompatible glass chip fabricated using a hybrid thin film and laminate technologies	0.735 μA μM^−1^ cm^−2^	Low O_2_ consumption on the electrode, long-term stability	[88]
Biocompatible PDMS biochip with Au/Nafion electrodes	50 mmol L^−1^	real-time and continuous O_2_ monitoring in dynamic flow conditions	[89]
Kapton tape with embedded spirally rolled Microchannels	12.89 nA mmHg ^−1^	O_2_ and temperature sensors, embedded spirally rolled microchannels	[90]
ElecCell technological platform using PVD	6 pA/s	low-cost, easy to use and reproducible portable chip	[91]
ultra-microelectrode array (UMEA)	0.49 nAs^−0.5^/mg/L	Ultra-short response time (<5 ms), 10 times lower O_2_ consumption	[92]
Multi-planar SPE sensor coupled with cultivation cell wells	3 mg/L	Continuous long-term O_2_ measurement, sensor reutilization	[93]
Inkjet printing (IJP) DO sensors on the delicate porous substrate	28 ± 1 nA L mg^−1^	low O_2_ consumption on electrodes, short response time (60 s)	[94]
Electrochemical microsensors combined with spheroid technology	NM	fast, precise, and continuous long-term measurement of metabolic directly in the microwell	[95]
Spheroid on chip	NM	Real-time monitoring of metabolic activity and automated assays for toxicity evaluation	[96]

NM: not mentioned.

## Data Availability

Not applicable.
